# Aluminates with Fluorinated Schiff Bases: Influence of the Alkali Metal–Fluorine Interactions in Structure Stabilization

**DOI:** 10.3390/molecules23123108

**Published:** 2018-11-27

**Authors:** Francisco M. García-Valle, Vanessa Tabernero, Tomás Cuenca, Jesús Cano, Marta E. G. Mosquera

**Affiliations:** Departamento de Química Orgánica y Química Inorgánica, Instituto de Investigacion en Química “Andres M. del Río” (IQAR) Universidad de Alcalá, Campus Universitario, 28871-Alcala de Henares, Madrid, Spain; blitzkrieg_bf109@yahoo.es (F.M.G.-V.); Vanessa.tabernero@uah.es (V.T.); tomas.cuenca@uah.es (T.C.)

**Keywords:** aluminium, alkali metals, Schiff bases, heterometallic compounds

## Abstract

New heterometallic aluminium-alkali metal compounds have been prepared using Schiff bases with electron withdrawing substituents as ligands. The synthesis of these new species was achieved via the reaction of AlMe_3_ with the freshly prepared alkali-metallated ligand. The derivatives formed were characterized by NMR in solution and by single crystal X-ray diffraction in the solid state. Aluminate derivatives with lithium and sodium were prepared and a clear influence of the alkali metal in the final outcome is observed. The presence of a Na···F interaction in the solid state has a stabilization effect and the species [NaAlMe_3_L]_2_ can de isolated for the first time, which was not possible when using Schiff bases without electron withdrawing substituents as ligands.

## 1. Introduction

Heterometallic compounds containing main group metals have generated great interest particularly in recent years [1,2,3,4,5]. Within those, -*ate* species are a remarkable class of compounds formed by two metals of very different natures, an electropositive alkali metal and a less electropositive one, very often a p-block metal [6,7,8,9,10,11]. These species not only show unusual structures, they also display a wide-ranging reactivity which, on many occasions, is diverse from the one shown by the homometallic analogous [12,13,14,15]. As such, these compounds have become very popular reagents for reactions such as the activation of unreactive C-H bonds [16,17,18], direct orthometalation processes [19,20,21] or the formation of C-C and C-heteroatom bonds [22,23,24,25]. They are also active catalysts for the polymerization of polar alkenes [26,27,28,29]. More recently, their activity in catalytic processes such as the hydroboration process [30] or Meerwein-Ponndorf-Verley (MPV) reactions [31] has also been described.

The type of ligands used for these -*ate* derivatives is relatively small, especially considering the significant potential that these compounds can display. In most cases, the reported species contains N-donor connectors [32,33,34,35], being less common O-donor linkers. Ligands with O- and N- donor groups are also scarce in these compounds. In our group, we described aluminate derivatives with functionalized phenolates as bridging ligands bearing donor substituents in *ortho* positions [36,37,38], and more recently, with terpene oximate ligands with O- and N- donor atoms [39].

Schiff bases or the phenoxo-imino ligands are a particularly popular type of O- and N-donor ligand. Many compounds containing them have been described that have shown a remarkable catalytic activity, particularly in polymerization processes [40,41,42,43]. There are quite a few examples of aluminium species with Schiff bases; however, no heterometallic derivatives had been reported, only recently we have published the first examples of aluminates with phenoxo-imino ligands, expanding the library of connecting ligands for -*ate* compounds [44].

The interest in phenoxo-imino ligands lies in the fact that they are very versatile and straightforward to prepare. By modifying the substituents, the electronic and steric properties of the compounds can be easily tuned [45,46,47,48]. In our previous study, we explored ligands bearing donor substituents in the iminic ring. In this work, we have extended the investigations to ligands with electron withdrawing substituents to analyse their effect on the final compounds. Interestingly, the introduction of fluorine atoms and the study of their influence on the nature of the synthesized compounds have not usually been reported for phenoxo-imino ligands [49]. As such, even though titanium compounds have been described with remarkable properties as catalysts for living ethylene polymerization [50] and Pd(II) complexes have shown to be active in hydrogenation processes [51], only 46 compounds with fluorinated phenoxo-imino ligands have been structurally characterized, mainly for titanium and aluminium [52].

In this paper, we report on the synthesis and characterization of the first examples of aluminates bearing fluorinated Schiff bases as ligands, completing the family of heterometallic aluminium-alkali metal compounds previously described by us [44]. For these species, the influence of the alkali metal is observed as well as the effect of the fluorine atoms located in the iminic ring of the ligand.

## 2. Results

The selected ligand precursor was prepared following the standard procedure for this kind of compound. In the present study, we chose the proligand HL*a* that incorporates fluorine atoms in positions 2 and 3 of the iminic ring [45,53,54].

**Scheme 1:**
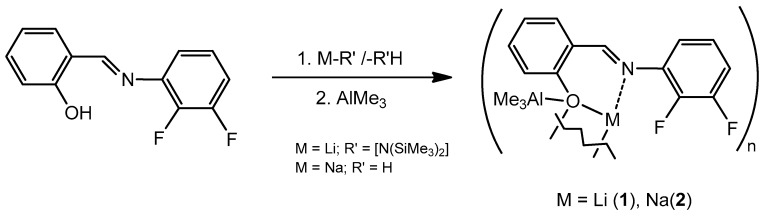
Synthesis of the alkali metal aluminate complexes [MAlMe_3_L*a*]_n_ (**1**–**2**).

Heterometallic complexes can be obtained following different reaction pathways. In this case, the procedure employed was the formation of the alkali metal complex in situ followed by the addition of the aluminium precursor. The stoichiometric reaction between the alkali metal precursors, [Li{N(SiMe_3_)_2_}] or NaH, and the proligand gave the alkali metal homometallic compounds. The subsequent addition of a stoichiometric amount of AlMe_3_ at low temperature led to the formation of the alkali metal aluminate complexes [MAlMe_3_(L*a*)] (M = Li, Na) (**1**–**2**), as shown in Scheme 1. These heterometallic complexes were characterized in the solid state by elemental analysis and by single crystal X-ray diffraction.

**Figure 1:**
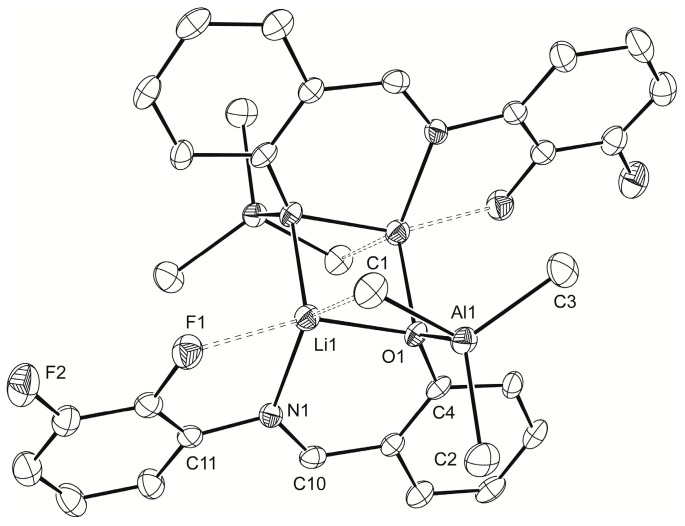
ORTEP plots for **1** showing thermal ellipsoids plots (30% probability). Hydrogen atoms are omitted for clarity. Selected bond distances (Å) and angles (°): C(1)-Al(1) 1.994(5), C(2)-Al(1) 1.953(6), C(3)-Al(1) 1.954(5), O(1)-Al(1) 1.902(3), C(1)···Li(1) 2.413(4), C(10)-N(1) 1.280(6), Li(1)-O(1) 1.982(8), Li(1)-O(1)#1 1.998(8), Li(1)-N(1) 2.007(9), Li(1)···F(1) 2.299(8), C(16)-F(1) 1.359(6), C(15)-F(2) 1.336(6), O(1)-Li(1)-O(1)#1 97.3(3), C(10)-N(1)-C(11) 118.5(4).

**Figure 2:**
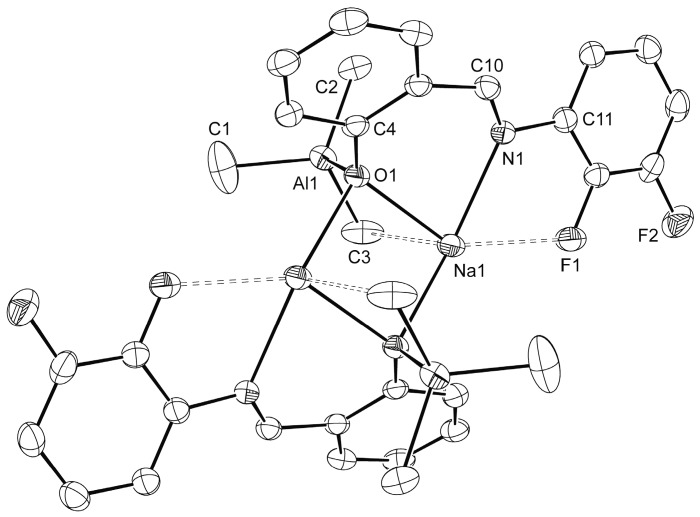
ORTEP plots for **2** showing thermal ellipsoids plots (30% probability). Hydrogen atoms are omitted for clarity. Selected bond distances (Å) and angles (deg.): C(3)-Al(1) 1.981(4), C(2)-Al(1) 1.965(3), C(1)-Al(1) 1.977(4), O(1)-Al(1) 1.8621(19), C(3)···Na(1) 2.737(6), C(10)-N(1) 1.275(3), Na(1)-O(1) 2.3254(18), Na(1)-O(1)#1 2.3657(19), Na(1)-N(1) 2.389(2), Na(1)-F(1) 2.4608(18), C(16)-F(1) 1.357(3), C(15)-F(2) 1.347(3), O(1)-Na(1)-O(1)#1 89.41(6), C(10)-N(1)-C(11) 117.3(2).

The single crystal X-ray diffraction studies allowed the unequivocal characterization in the solid state of compounds **1** and **2**. As shown in Figure 1 and Figure 2, both the lithium and sodium are tetranuclear species M_2_Al_2_ (M = Li, Na). The phenoxo-imino ligand acts as a bridge between the aluminium and the alkali metal through the oxygen atom. The ligand also links the alkali metals through the oxygen and a M_2_O_2_ (M = Li, Na) central core is observed. Although **1** and **2** are isostructural molecules some differences in the orientation of the ligands are detected, as such, the rings from the phenoxo-imino ligand show a significantly more coplanar disposition for the lithium compound **1** (angle 16.68°) than for **2** (angle 45.18°). The Al-O distance is significantly shorter for the sodium derivative **2** (1.900(3) Å **1** vs. 1.8621(19) Å **2**), which could be related to the fact that a higher ionic component in the M-O bonding for the sodium compound can be expected, which then may provoke a stronger interaction of the aluminium and the phenoxo oxygen. Finally, the AlMe_3_ moiety is connected to the ligand as if these compounds have been generated from the breakage of the (AlMe_3_)_2_ precursor by an O-donor species, such as the metallated ligand, to form a 1:1 Lewis adduct.

In both compounds, the alkali metal shows a pentacoordinated environment. As such, besides the oxygen atom, the iminic nitrogen atom also establishes a donor interaction with alkali metal and one of the methyl groups bonded to the aluminium interacts with the alkali metal through a M···Me contact. The M···C distances (2.413(4) Å for **1** and 2.737(6) Å for **2**) are within the shortest found in the literature [44,52]. Interestingly, the longest Al-C distance belongs to the methyl group involved in the Me···M interaction (1.994(5) Å for **1** and 1.981(4) Å for **2**). These contacts contribute to the stabilization of the alkali metal coordination sphere. Furthermore, the *ortho* fluorine atom of the iminic ring establishes a M···F interaction (2.299(8) Å for **1** and 2.4608(18) Å for **2**) that completes the unusual pentacoordinated sphere for these metallic centres. Of particular interest is the presence of Na···F contacts since this represents a stabilizing interaction that allows the isolation of this compound. As such, for the species previously reported by us without fluorine substituents in the iminic ring, the analogous [NaAlMe_3_(L)] compound was not possible to detect or isolate. Compound **2** is then the first example of a sodium aluminate of stoichiometry [NaAlMe_3_(L)]_n_ with phenoxo-imino ligands and one of the very few reported. The influence of Na···F interactions has been acknowledged not only in the structure but also in the reactivity of heterometallic sodium species as it has been reported for metallated reactions [55].

Finally, an interesting feature in the packing of **2**, is the presence of π−π stacking interactions between the fluorinated rings, which are oriented with an anti-disposition of each other (considering the fluorine substituents), the distance between centroids is 3.62 Å, and directs the packing into chains along the *c* axis.

**Figure 3:**
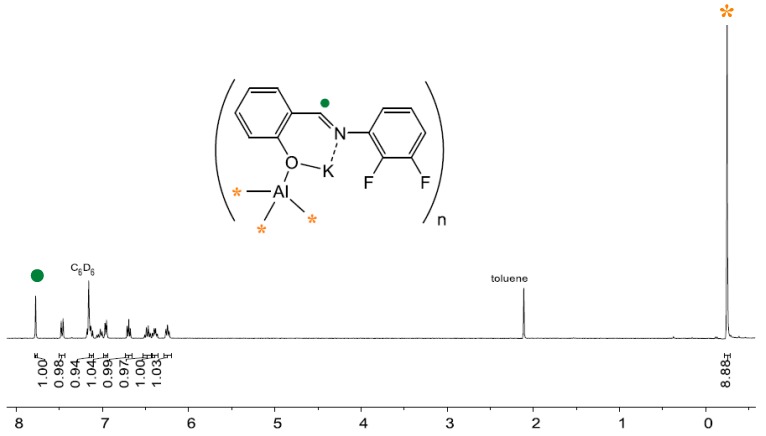
^1^H NMR spectrum of complex **1** recorded in C_6_D_6_ at room temperature.

**Figure 4:**
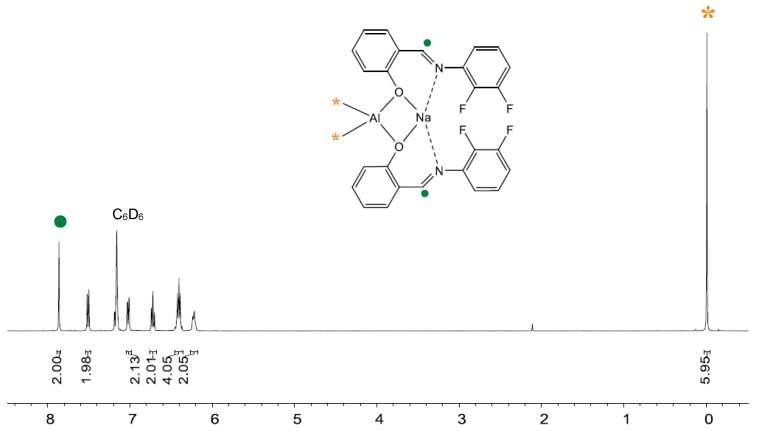
^1^H NMR spectrum of complex **3** recorded in C_6_D_6_ at room temperature.

These species were also characterized in solution by multinuclear NMR spectroscopy (Figure 3 and Figure 4). ^1^H NMR spectra were recorded in C_6_D_6_ and displayed the resonances for the methyl groups bound to the aluminium centre at negative values. For **1** appears at *δ*−0.24 ppm and integrate for three methyl groups. These data suggest that the aluminate species show the expected [LiAlMe_3_(L)]_n_ formulation also in solution. In comparison to the analogous species without the fluorine substituents, the methyl groups appear at a lower field, indicating a more acidic character, as could be expected due to the presence of these electron withdrawing groups atoms in the iminic ring [44].

**Scheme 2:**
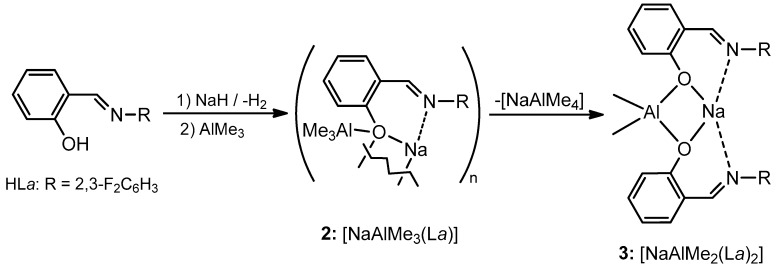
Proposed reaction for the formation of [NaAlMe_2_(L)_2_] (**3**).

However, for compound **2**, in the NMR different behaviour was observed (Figure 4). In this case, once the crystals of **2** were dissolved, the ^1^H NMR spectrum in benzene-*d*_6_ did not reveal the expected resonances for the characterized aluminate in the solid state. The signal of the methyl groups bound to the aluminium centre appeared at δ−0.01 ppm, a remarkable shift compared with the lithium aluminate; moreover, this signal is consistent with three hydrogen atoms per ligand in agreement with a [NaAlMe_2_(L*a*)_2_] (**3**) formulation (Scheme 2), a disposition already described in our previous work for the phenoxo-imino species with donor substituents [44]. In fact, for those species without the fluorine substituents, it was not possible to isolate the analogous sodium derivate, as it evolves very rapidly into the formation of compounds with the [NaAlMe_2_L_2_] formulation, via a rearrangement process that also might generate [NaAlMe_4_], as shown in Scheme 2. In this case, the presence of the Na···F interactions seems to stabilize the structure and it was possible to isolate [NaAlMe_3_L*a*] in the solid state.

**Scheme 3:**
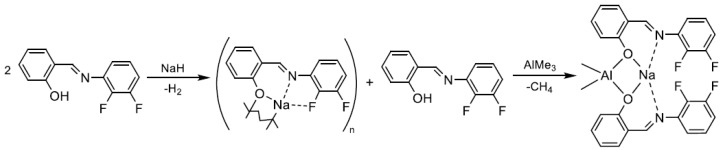
Synthesis of [NaAlMe_2_(L*a*)_2_] (**3**).

Taking into account these data, different reactions were carried out to understand the nature of this process. In the first place, the reaction with the correct stoichiometry (2:1:1 ratio of [HL*a*]:[Na]:[Al]) to obtain the complex **3**, [NaAlMe_2_(L*a*)_2_], was performed (Figure S1). In this case, after the addition of the alkali metal precursor, a mixture of the proligand and the metallated ligand was formed, and the subsequent incorporation of AlMe_3_ gave the aluminate complex **3**, [NaAlMe_2_(L*a*)_2_], which displayed an identical ^1^H NMR spectrum as the one observed for the isolated crystals (Scheme 3).

Besides, to support the proposal where the reaction pathway follows a ligand rearrangement with the formation of sodium tetramethylaluminate, [NaAlMe_4_], the stoichiometric reaction (1:1:1 of [HL*a*]:[Na]:[Al]) was monitored in a valved NMR tube. In this reaction, a signal at δ-0.40 ppm was detected that can be attributed to the tetramethylaluminate species [NaAlMe_4_] in agreement with the mechanism proposed (Figure S2). We had already observed this behaviour in the formation of [NaAlMe_2_(L)_2_] aluminates with phenoxo-imino ligands containing donor substituents [44].

**Scheme 4:**
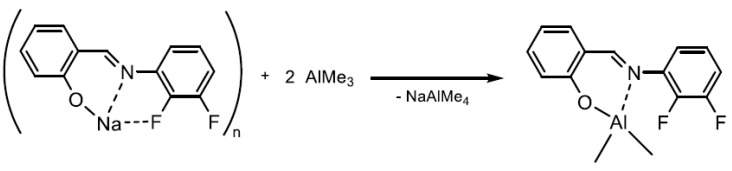
Metathesis reaction between the sodium complex and AlMe_3_.

Finally, to have clear evidence regarding the formation of [NaAlMe_4_], the reaction between the alkali metal compound [NaL*a*] with two equivalents of trimethylaluminium (AlMe_3_) was performed (Scheme 4) [56]. In this reaction, the aluminium derivative [AlMe_2_(L*a*)] was clearly identified, which proves the possibility of ligand rearrangements in these systems. Confirming this, a signal at δ-0.40 ppm attributable to [NaAlMe_4_] could also be detected (Figure S3).

## 3. Materials and Methods

### 3.1. General Procedures

All manipulations were performed under an inert atmosphere using Schlenk-line techniques (O_2_ < 3 ppm) and a glove box (O_2_ < 0.6 ppm) MBraun MB-20G (MBraun, Garching, Germany). Solvents were purified using an MBraun Solvent Purification System (MBraun, Garching, Germany). Deuterated solvents were degassed and stored in the glove box in the presence of molecular sieves (4 Å). Fluoroaniline compounds were purchased from Fluorochem (Fluorochem, Derbyshire, UK) and used as received. 2-hydroxybenzaldehyde and metallic precursors were purchased from Sigma-Aldrich (Merck, Darmstadt, Germany). NMR spectra were recorded with a Bruker 400 Ultrashield (Bruker, Karlsruhe, Germany) (^1^H 400 MHz, ^13^C 101 MHz) at room temperature. All chemical shifts were determined using the residual signal of solvents and were reported versus SiMe_4_. The assignment of the signal was carried out from 1D (^1^H, ^19^F{^1^H}, ^13^C{^1^H}) and 2D (^1^H-^13^C HSQC) NMR experiments. Elemental analyses were performed with a PerkinElmer 2400 CHNS/O analyzer Series II (PerkinElmer, Ohio, OH, USA) and were the average of at least two independent measurements.

### 3.2. Synthesis of Complex [LiAlMe_3_(O-2-{2,3-C_6_H_3_F_2_N=CH}C_6_H_4_)], [LiAlMe_3_La] (***1***)

At room temperature, a mixture of HL*a* (0.700 g, 3.00 mmol) and [Li{N(SiMe_3_)_2_}] (0.518 g, 3.60 mmol) in toluene (30 mL) was stirred for one night. The resultant solution was cooled to −78 °C, and AlMe_3_, 2 M in toluene (1.50 mL, 3.00 mmol) was added dropwise. Then, the reaction was warmed to room temperature, and it was concentrated to 10 mL and stored at −20 °C. After a few days, crystals were obtained. Yield: 0.251 g, 27%. The single crystal used for the for X-ray diffraction analysis were obtained from a NMR tube. ^1^H NMR (C_6_D_6_, 400 MHz, 295 K): δ 7.78 (s, 1H, *H*C=N), 7.47 (d, 1H, C_6_*H*_4_), 7.15 (m, 1H, Ar*H*), 6.96 (m, 1H, Ar*H*), 6.69 (m, 1H, Ar*H*), 6.47 (m, 1H, Ar*H*), 6.39 (m, 1H, Ar*H*), 6.23 (m, 1H, Ar*H*), −0.24 [s, 9H, Al(C*H*_3_)_3_]. ^13^C NMR (C_6_D_6_, 101 MHz, 295 K): δ 164.1 (*C*=N), 161.9 (*C*-O), multiplets from 151.9 to 144.5 (*C*-F, hardly assignable due to complicated ^13^C-^19^F coupling), 136.5, 136.0, 124.8, 122.3, 122.1, 118.7, 115.1, 114.9, 112.1 (Ar-*C*), −8.14 [Al(*C*H_3_)_3_]. ^19^F NMR (C_6_D_6_, 376 MHz, 295 K): δ-136.7 (d, 1F, *o*-F), −154.8 (d, 1F, *m*-F). Anal. Calcd for C_16_H_17_AlF_2_LiNO (311.24 g/mol): C 61.75, H 5.51, N 4.50. Found: C 61.62, H 5.21, N 4.96.

### 3.3. Synthesis of Complex [NaAlMe_3_{(O-2-(2,3-C_6_H_3_F_2_N=CH)C_6_H_4_)}], [NaAlMe_3_(La)] (***2***)

The same method as that for **1** was used but with **HL*a*** (0.560 g, 2.40 mmol), NaH (0.057 g, 2.40 mmol) and AlMe_3_, 2 M in toluene (1.20 mL, 2.40 mmol). Yield: 0.221 g, 27%. Anal. Calcd for C_16_H_17_AlF_2_NaNO (327.28 g/mol): C 58.72, H 5.24, N 4.28. Found: C 59.31, H 5.18, N 4.54.

### 3.4. Synthesis of Complex [NaAlMe_2_{(O-2-(2,3-C_6_H_3_F_2_N=CH)C_6_H_4_)}_2_], [NaAlMe_2_(La)_2_] (***3***)

At room temperature, a mixture of **HL*a*** (0.560 g, 2.40 mmol) and NaH (0.029 g, 1.20 mmol) in toluene (20 mL) was stirred few hours. This solution was cooled to −78 °C, and AlMe_3_, 2 M in toluene (0.60 mL, 1.20 mmol) was added dropwise. Then, the mixture was warmed to room temperature, and reacted one night. After, the solution was dried under vacuum and the resultant solid was washed with n-hexane twice to give a yellow powder. Yield: 0.503 g, 77%. ^1^H NMR (C_6_D_6_, 400 MHz, 295 K): δ 7.86 (s, 2H, *H*C=N), 7.51 (d, 2H, C_6_*H*_4_), 7.19 (bs, 2H, Ar*H*), 7.02 (m, 2H, C_6_*H*_4_), 6.72 (m, 2H, Ar*H*), 6.42–6.21 (m, 6H, Ar*H*), −0.01 [s, 6H, Al(C*H*_3_)]. ^13^C NMR (C_6_D_6_, 101 MHz, 295 K): δ 168.3 (*C*-O), 162.4 (*C*=N), multiplets from 152.0 to 143.3 (*C*-F, hardly assignable due to complicated ^13^C-^19^F coupling), 142.0, 137.6, 134.8, 133.3, 124.6, 123.5, 122.5, 118.3, 115.0, 114.1 (Ar-*C*), −8.18 [Al(*C*H_3_)]. ^19^F NMR (C_6_D_6_, 376 MHz, 295 K): δ-137.9 (d, 1F, *o*-F), −157.1 (d, 1F, *m*-F). Anal. Calcd for C_28_H_22_AlF_4_N_2_NaO_2_ (544.46 g/mol): C 61.77, H 4.07, N 5.15. Found: C 61.47, H 4.18, N 5.40.

### 3.5. Single-crystal X-Ray Structure Determination for (***1***·2C_6_D_6_) and ***2*** (Table 1)

Data collection was performed at 200(2) K, with the crystals covered with perfluorinated ether oil. Single crystals of **1c** were mounted on a Bruker-Nonius Kappa CCD single crystal diffractometer equipped with a graphite-monochromated Mo-Kα radiation (λ = 0.71073 Å). Multiscan [57] absorption correction procedures were applied to the data. The structure was solved using the WINGX package [58], by direct methods (SHELXS-97) and refined using full-matrix least-squares against *F*^2^ (SHELXL-97) [59,60]. All non-hydrogen atoms were anisotropically refined. Hydrogen atoms were geometrically placed and left riding on their parent atoms except for the carbon atoms involved in the interaction with the alkali metal in **2** (C3), and for the iminic carbon in **1** (C10), those atoms were found in the Fourier map and refined freely. For **1** disordered solvent molecules were present in the asymmetric unit: two molecules of benzene per molecule of **1**. No chemical sense could be made of the disorder solvent molecule, so a squeeze procedure [61,62] was applied to remove its contribution from the structure factors. Full-matrix least-squares refinements were carried out by minimizing ∑w(*Fo*^2^ − *Fc*^2^)^2^ with the SHELXL-97 weighting scheme and stopped at shift/err < 0.001. The final residual electron density maps showed no remarkable features. Crystallographic data for the structure reported in this paper have been deposited with the Cambridge Crystallographic Data Centre as supplementary publication no. CCDC-1878166(**1·**2C_6_D_6_) and CCDC-1878167(**2**).

## 4. Conclusions

For the first time, the synthesis of alkali metal aluminates [MAlMe_3_(L*a*)] (M = Li (**1**), Na (**2**)) has been achieved with fluorinated Schiff bases as ligands. The presence of the fluorine substituents in the ligands facilitates the isolation of the aluminate [NaAlMe_3_(L*a*)] (**2**) in the solid state thanks to the presence of a stabilizing Na···F interaction, in contrast with the behaviour observed in analogous compounds without fluorine substituents in the iminic ring. Although the lithium derivative **1** maintains its structure when dissolved, the sodium compound **2** in the solution evolves rapidly into the formation of [MAlMe_3_(L*a*)]_2_. The mechanism for this transformation is based on interchange reactions via a ligand rearrangement with the formation of the [MAlMe_4_] species, which can be detected by NMR techniques, in a similar way as observed previously for the aluminates without fluorine substituents. Studies of the reactivity of these species towards small molecules are ongoing.

## Figures and Tables

**Table 1:** Crystallographic data for **1·**2C_6_D_6_, and **2**.

	[LiAlMe_3_L*a*]·2C_6_D_6_	[NaAlMe_3_L*a*]
Empirical formula	C_32_H_34_Al_2_Li_2_F_4_N_2_O_2_·2C_6_D_6_	C_32_H_34_Al_2_Na_2_F_4_N_2_O_2_
Formula weight	790.59	654.55
Colour, shape	Yellow/block	Yellow/block
Crystal size (mm)	0.45 × 0.42 × 0.27	0.49 × 0.48 × 0.45
Crystal system	Monoclinic	Triclinic
Space group	*P2_1_/c*	*P-1*
a (Å)	11.699(3)	8.5668(8)
b (Å)	16.669(6)	10.2749(8)
c (Å)	10.7558(17)	11.1625(8)
α (°)	90	111.026(6)
β (°)	90.35(2)	91.093(5)
γ (°)	90	112.001(6)
V (Å^3^)	2097.5(9)	836.87(13)
Z	2	1
ρ_calcd._ (mg m^−3^)	1.233	1.299
F_000_	816	340
μ (mm^−1^)	0.125	0.166
*θ* Range (°)	3.001 to 27.518	3.063 to 27.498
Reflns. Collected	16768	7166
Indep. Reflns./R(int)	4744/0.2050	3829/0.0734
Data/restraints/param	4744/147/203	3829/0/215
*R_1_/wR_2_* (I > 2*σ*(I)) *^a^*	0.0982/0.2357	0.0498/0.1165
*R_1_/wR_2_* (all data)*^a^*	0.1892/0.2927	0.1230/0.1505
GOF	0.873	0.918
Max/min Δρ (e.Å^−3^)	0.524 and −0.931	0.238 and −0.473

^a^*R_1_* = Σ(||*F*_o_| − |*F*_c_||)/Σ|*F*_o_|; *wR_2_* = {Σ[*w*(*F*_o_^2^ − *F*_c_^2^)^2^]/Σ[*w*(*F*_o_^2^)^2^]}^1/2^; GOF = {Σ[*w*(*F*_o_^2^ − *F*_c_^2^)^2^]/(n − p)}^1/2.^

## Supplementary Materials

The following are available online at http://www.mdpi.com/1420-3049/23/12/3108/s1.

## References

[1] García-Alvarez J., Hevia E., Kennedy A.R., Klett J., Mulvey R.E. (2007). Lewis base stabilized lithium TMP-aluminates: An unexpected fragmentation and capture reaction involving cyclic ether 1,4-dioxane. Chem. Commun..

[2] Delferro M., Marks T.J. (2011). Multinuclear olefin polymerization catalysts. Chem. Rev..

[3] Mandal S.K., Roesky H.W. (2010). Assembling heterometals through oxygen: An efficient way to design homogeneous catalysts. Acc. Chem. Res..

[4] Mulvey R.E. (2001). s-Block metal inverse crowns: Synthetic and structural synergism in mixed alkali metal-magnesium (or zinc) amide chemistry. Chem. Commun..

[5] Kennedy A.R., Klett J., Mulvey R.E., Wright D.S. (2009). Synergic sedation of sensitive anions: Alkali-mediated zincation of cyclic ethers and ethene. Science.

[6] Harrison-Marchand A., Mongin F. (2013). Mixed AggregAte (MAA): A single concept for all dipolar organometallic aggregates. 1. Structural Data. Chem. Rev..

[7] Wheatley A.E.H. (2004). Recent developments in the synthetic and structural chemistry of lithium zincates. New. J. Chem..

[8] Conway B., Crosbie E., Kennedy A.R., Mulvey R.E., Robertson S.D. (2012). Regioselective heterohalogenation of 4-halo-anisoles via a series of sequential ortho-aluminations and electrophilic halogenations. Chem. Commun..

[9] Wittig G., Meyer F.J., Lange G. (1951). Über das Verhalten von Diphenylmetallen als Komplexbildner. Justus Liebigs Ann. Chem..

[10] Wittig G. (1958). Komplexbildung und Reaktivität in der metallorganischen Chemie. Angew. Chem..

[11] Mulvey R.E., Robertson S.D. (2014). FascinATES: Mixed-metal ate compounds that function synergistically. Top. Organomet. Chem..

[12] Mulvey R.E. (2006). Modern ate chemistry:  Applications of synergic mixed alkali-metal-magnesium or -Zinc reagents in synthesis and structure building. Organometallics.

[13] Greer J.A., Blair V.L., Thompson C.D., Andrews P.C. (2016). Simplifying metal-‘ate’ chemistry: Formation and comprehensive characterisation of a homo-metallic amido lithiate complex. Dalton Trans..

[14] Mongin F., Harrison-Marchand A. (2013). Mixed AggregAte (MAA): A single concept for all Dipolar organometallic aggregates. 2. Syntheses and Reactivities of Homo/HeteroMAAs. Chem. Rev..

[15] Hatano M., Yamashita K., Mizuno M., Ito O., Ishihara K. (2015). C-Selective and Diastereoselective Alkyl Addition to beta, gamma-Alkynyl-alpha-imino Esters with Zinc(II)ate Complexes. Angew. Chem. Int. Ed..

[16] Mulvey R.E., Blair V.L., Clegg W., Kennedy A.R., Klett J., Russo L. (2010). Cleave and capture chemistry illustrated through bimetallic-induced fragmentation of tetrahydrofuran. Nat. Chem..

[17] Mulvey R.E., Mongin F., Uchiyama M., Kondo Y. (2007). Deprotonative Metalation Using Ate Compounds: Synergy, Synthesis, and Structure Building. Angew. Chem. Int. Ed..

[18] Uzelac M., Hevia E. (2018). Polar organometallic strategies for regioselective C–H metallation of N-heterocyclic carbenes. Chem. Commun..

[19] Naka H., Uchiyama M., Matsumoto Y., Wheatley A.E.H., McPartlin M., Morey J.V., Kondo Y. (2007). An Aluminum Ate Base:  Its Design, Structure, Function, and Reaction Mechanism. J. Am. Chem. Soc..

[20] Uchiyama M., Naka H., Matsumoto Y., Ohwada T. (2004). Regio-and chemoselective direct generation of functionalized aromatic aluminum compounds using aluminum ate base. J. Am. Chem. Soc..

[21] Naka H., Morey J.V., Haywood J., Eisler D.J., McPartlin M., Garcia F., Kudo H., Kondo Y., Uchiyama M., Wheatley A.E.H. (2008). Mixed Alkylamido Aluminate as a Kinetically Controlled Base. J. Am. Chem. Soc..

[22] Krasovskiy A., Knochel P. (2004). A LiCl-Mediated Br/Mg Exchange Reaction for the Preparation of Functionalized Aryl- and Heteroarylmagnesium Compounds from Organic Bromides. Angew. Chem. Int. Ed..

[23] Haag B., Mosrin M., Ila H., Malakhov V., Knochel P. (2011). Regio- and Chemoselective Metalation of Arenes and Heteroarenes Using Hindered Metal Amide Bases. Angew. Chem. Int. Ed..

[24] Hevia E., Chua J.Z., Garcia Alvarez P., Kennedy A.R., McCall M.D. (2010). Exposing the hidden complexity of stoichiometric and catalytic metathesis reactions by elucidation of Mg-Zn hybrids. Proc. Natl. Acad. Sci..

[25] Davin L., McLellan R., Kennedy A.R., Hevia E. (2017). Ligand-induced reactivity of [small beta]-diketiminate magnesium complexes for regioselective functionalization of fluoroarenes via C-H or C-F bond activations. Chem. Commun..

[26] Rodriguez-Delgado A., Chen E.Y. (2005). Single-Site Anionic Polymerization. Monomeric Ester Enolaluminate Propagator Synthesis, Molecular Structure, and Polymerization Mechanism. J. Am. Chem. Soc..

[27] Casey C., Case M.C., Shusterman A.J. (2012). Stereochemistry and Mechanism of the Ring-Opening Reaction of Cyclopropylenones with LiCu(Me)_2_. Organometallics.

[28] Harvey M.J., Proffitt M., Wei P., Atwood D.A. (2001). Monomeric uni-ligated aluminates. Chem. Commun..

[29] Singh S., Chai J., Pal A., Jancik V., Roesky H.W., Herbst-Irmer R. (2007). Base free lithium-organoaluminate and the gallium congener: Potential precursors to heterometallic assemblies. Chem. Commun..

[30] Pollard V.A., Orr S.A., McLellan R., Kennedy A.R., Hevia E., Mulvey R.E. (2018). Lithium diamidodihydridoaluminates: Bimetallic cooperativity in catalytic hydroboration and metallation applications. Chem. Commun..

[31] Hua Y., Guo Z., Han H., Wei X. (2017). N,N,O-Tridentate Mixed Lithium–Magnesium and Lithium–Aluminum Complexes: Synthesis, Characterization, and Catalytic Activities. Organometallics.

[32] Amstrong D.R., Crosbie E., Hevia E., Mulvey R.E., Ramsay D.L., Robertson S.D. (2014). TMP (2,2,6,6-tetramethylpiperidide)-aluminate bases: Lithium-mediated alumination or lithiation-alkylaluminium-trapping reagents?. Chem. Sci..

[33] Mulvey R.E., Amstrong D.R., Conway B., Crosbie E., Kennedy A.R., Robertson S.D. (2011). Structurally Powered Synergic 2,2,6,6-Tetramethylpiperidine Bimetallics: New Reflections through Lithium-Mediated Ortho Aluminations. Inorg. Chem..

[34] Crosbie E., García-Álvarez P., Kennedy A.R., Klett J., Mulvey R.E., Robertson S.D. (2010). Structurally Engineered Deprotonation/Alumination of THF and THTP with Retention of Their Cycloanionic Structures. Angew. Chem. Int. Ed..

[35] Rohbogner C.J., Wunderlich S.H., Clososki G.C., Knochel P. (2009). New Mixed Li/Mg and Li/Mg/Zn Amides for the Chemoselective Metallation of Arenes and Heteroarenes. Eur. J. Org. Chem..

[36] Muñoz M.T., Urbaneja C., Temprado M., Mosquera M.E.G., Cuenca T. (2011). Lewis acid fragmentation of a lithium aryloxide cage: Generation of new heterometallic aluminium-lithium species. Chem. Commun..

[37] Muñoz M.T., Cuenca T., Mosquera M.E.G. (2014). Heterometallic aluminates: Alkali metals trapped by an aluminium aryloxide claw. Dalton Trans..

[38] Muñoz M.T., Barandika G., Bazán B., Cuenca T., Mosquera M.E.G. (2017). Aluminum Alkali Metalate Derivatives: Factors Driving the Final Nuclearity in the Crystal Form. Eur. J. Inorg. Chem..

[39] Fernández-Millán M., Temprado M., Cano J., Cuenca T., Mosquera M.E.G. (2016). Synthesis of novel chiral heterometallic terpene oximates: Unusual generation of an aluminium enolate by a cooperative effect. Dalton Trans..

[40] Zhang J., Jian C., Gao Y., Wang L., Tang N., Wu J. (2012). Synthesis and Characterization of Multi-Alkali-Metal Tetraphenolates and Application in Ring-Opening Polymerization of Lactide. Inorg. Chem..

[41] Zhang J., Xiong J., Sun Y., Tang N., Wu J. (2014). Highly Iso-Selective and Active Catalysts of Sodium and Potassium Monophenoxides Capped by a Crown Ether for the Ring-Opening Polymerization of rac-Lactide. Macromolecules.

[42] Nomura N., Ishii R., Yamamoto Y., Kondo T. (2007). Stereoselective ring-opening polymerization of a racemic lactide by using achiral salen- and homosalen-aluminum complexes. Chem.-Eur. J..

[43] Normand M., Dorcet V., Kirillov E., Carpentier J.-F. (2013). {Phenoxy-imine} aluminum versus-indium complexes for the immortal ROP of lactide: Different stereocontrol, different mechanisms. Organometallics.

[44] Garcia-Valle F.M., Tabernero V., Cuenca T., Cano J., Mosquera M.E.G. (2018). Schiff-base -ate derivatives with main group metals: Generation of a tripodal aluminate metalloligand. Dalton Trans..

[45] Makio H., Terao H., Iwashita A., Fujita T. (2011). FI Catalysts for Olefin Polymerization-A Comprehensive Treatment. Chem. Rev..

[46] Dagorne S., Janowska I., Welter R., Zakrzewski J., Jaouen G. (2004). Synthesis and Structure of a Four-Coordinate Aluminum Alkyl Cation/HB(C6F5)3 Salt:  Implication in a B(C6F5)3-Catalyzed Hydroalumination Reaction of Benzophenone or Benzaldehyde. Organometallics.

[47] Martínez G., Cuenca T., Mosquera M.E.G. (2012). Effect of the Nitrogen Substituent on the Reactions of Alane towards Imino- and Aminophenols: Generation of a Dinuclear Aluminoxane. Eur. J. Inorg. Chem..

[48] García-Valle F.M., Estivill R., Gallegos C., Cuenca T., Mosquera M.E.G., Tabernero V., Cano J. (2015). Metal and Ligand-Substituent Effects in the Immortal Polymerization of rac-Lactide with Li, Na, and K Phenoxo-imine Complexes. Organometallics.

[49] Iwasa N., Katao S., Liu J., Fujiki M., Furukawa Y., Nomura K. (2009). Notable Effect of Fluoro Substituents in the Imino Group in Ring-Opening Polymerization of ε-Caprolactone by Al Complexes Containing Phenoxyimine Ligands. Organometallics.

[50] Saito J., Mitani M., Mohri J.-I., Yoshida Y., Matsui S., Ishii S.-I., Kojoh S.-I., Kashiwas N., Fujita T. (2001). Living Polymerization of Ethylene with a Titanium Complex Containing Two Phenoxy-Imine Chelate Ligands. Angew. Chem. Int. Ed..

[51] Kasumov V.T., Uçar I., Bulut A. (2010). Synthesis, structural, spectroscopic and reactivity properties of a new N-2,3,4-trifluorophenyl-3,5-di-tert-butylsalicylaldimine ligand and its Cu(II) and Pd(II) complexes. J. Fluorine Chem..

[52] Cambridge Structural Database (CSD version 5.39, May 2018). https://www.ccdc.cam.ac.uk/support-and-resources/ccdcresources/csd-2018-updates/.

[53] Pang W., Zhao J.-W., Zhao L., Zhang Z.-K., Zhu S.-Z. (2015). Synthesis, characterization and comparative study of a series of fluorinated Schiff bases containing different orientation CHN spacers. J. Mol. Struct..

[54] García-Valle F.M., Tabernero V., Cuenca T., Mosquera M.E.G., Cano J., Milione S. (2018). Biodegradable PHB from rac-β-Butyrolactone: Highly Controlled ROP Mediated by a Pentacoordinated Aluminum Complex. Organometallics.

[55] Maddock L.C.H., Nixon T., Kennedy A.R., Hevia E., Probert M.R., Clegg W. (2018). Utilising Sodium-Mediated Ferration for Regioselective Functionalisation of Fluoroarenes via C-H and C-F Bond Activations. Angew. Chem. Int. Ed..

[56] Kaneko H., Dietrich H.M., Schadle C., Maichle-Mossmer C., Tsurugi H., Tornroos K.W., Mashima K., Anwander R. (2013). Synthesis of Rare-Earth-Metal Iminopyrrolyl Complexes from Alkyl Precursors: Ln→Al N-Ancillary Ligand Transfer. Organometallics.

[57] Blessing R.H. (1995). An empirical correction for absorption anisotropy. Acta Crystallogr. Sect. A.

[58] Farrugia L.J. (1999). WinGX suite for small-molecule single-crystal crystallography. J. Appl. Cryst..

[59] Sheldrick G.M. (2008). A short history of SHELX. Acta Crystallogr. Sect. A.

[60] Sheldrick G.M. (2015). Crystal structure refinement with SHELXL. Acta Crystallogr. Sect. C.

[61] Spek A. (2003). Single-crystal structure validation with the program PLATON. J. Appl. Cryst..

[62] Van der Sluis P., Spek A.L. (1990). BYPASS: An effective method for the refinement of crystal structures containing disordered solvent regions. Acta Crystallogr. Sect. A.

