# Supplementary material for: Aluminates with Fluorinated Schiff Bases: Influence of the Alkali Metal–Fluorine Interactions in Structure Stabilization
Source: Molecules. 2018 Nov 27;23(12):3108. doi: 10.3390/molecules23123108 (PMC6321257; doi:10.3390/molecules23123108)
Supplement: Supplementary file 1 [file molecules-23-03108-s001.zip › molecules-397442-sup-proof back/molecules-397442-sup-proof back.pdf]

Supporting Information for

Aluminates with Fluorinated Schiff Bases: Influence of the Alkali Metal–Fluorine Interaction in Structure Stabilization

Francisco M. García-Valle, Vanessa Tabernero, Tomás Cuenca, Jesús Cano,* and Marta E. G. Mosquera*[a]

[a] *Departamento de Química Orgánica y Química Inorgánica, Instituto de Investigación en Química "Andrés M. del Río" (IQAR), Universidad de Alcalá, Campus Universitario, 28871-Alcalá de Henares, Spain*

1. **^{1}H NMR spectrum for the reaction performed with a 2:1:1 ratio**
2. **^{1}H NMR spectrum for the reaction with a 1:1:1 ratio ([HL]/[M]/[Al]) monitored in a NMR tube**
3. **^{1}H NMR spectrum of [NaL*a*] and $AlMe_3$ reaction in a 1:2 molar ratio**
4. **Single-Crystal X-ray Structure Determination of ($1 \cdot 2C_6H_6$) and 2.**

1. ^{1}H NMR spectrum for the reaction performed with a 2:1:1 ratio

- Complex [NaAlMe2(L*a*)2], (**3**)

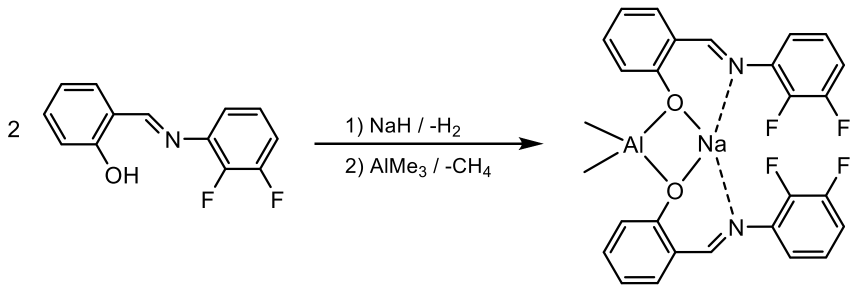

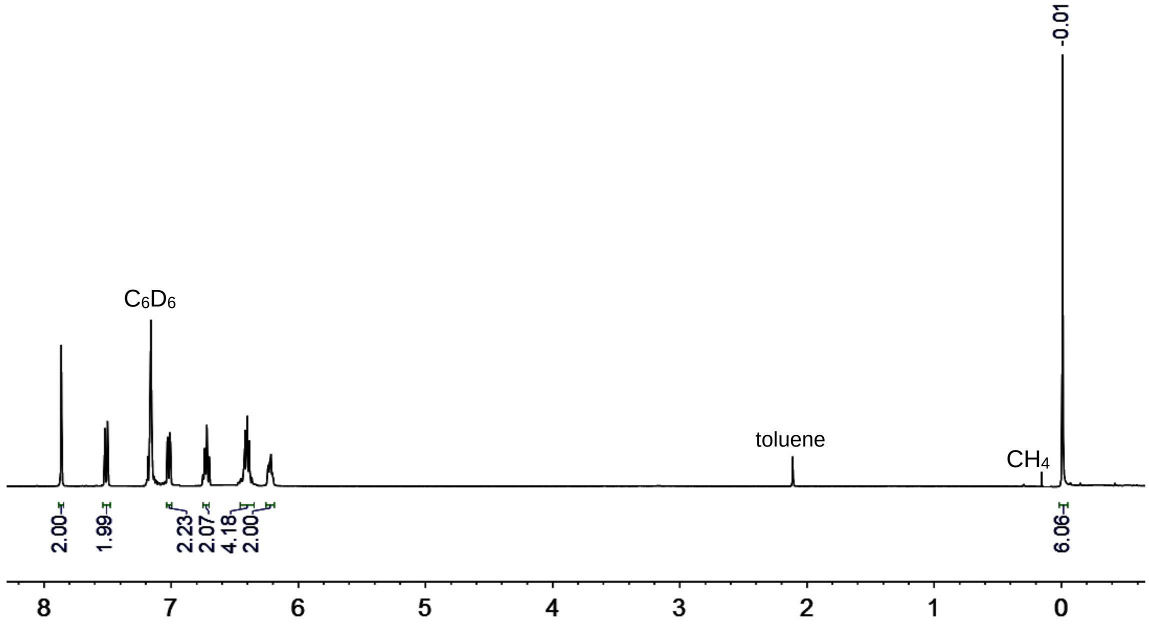

Figure S1 ^{1}H NMR spectrum of complex **3** obtained in the stoichiometric reaction, and recorded in C_6D_6 at room temperature.

2. ^{1}H NMR spectrum for the reaction with a 1:1:1 ratio ([HL]/[M]/[Al]) monitored in a NMR tube

- Complex [NaAlMe$_2$(L*a*)$_2$], (**3**)

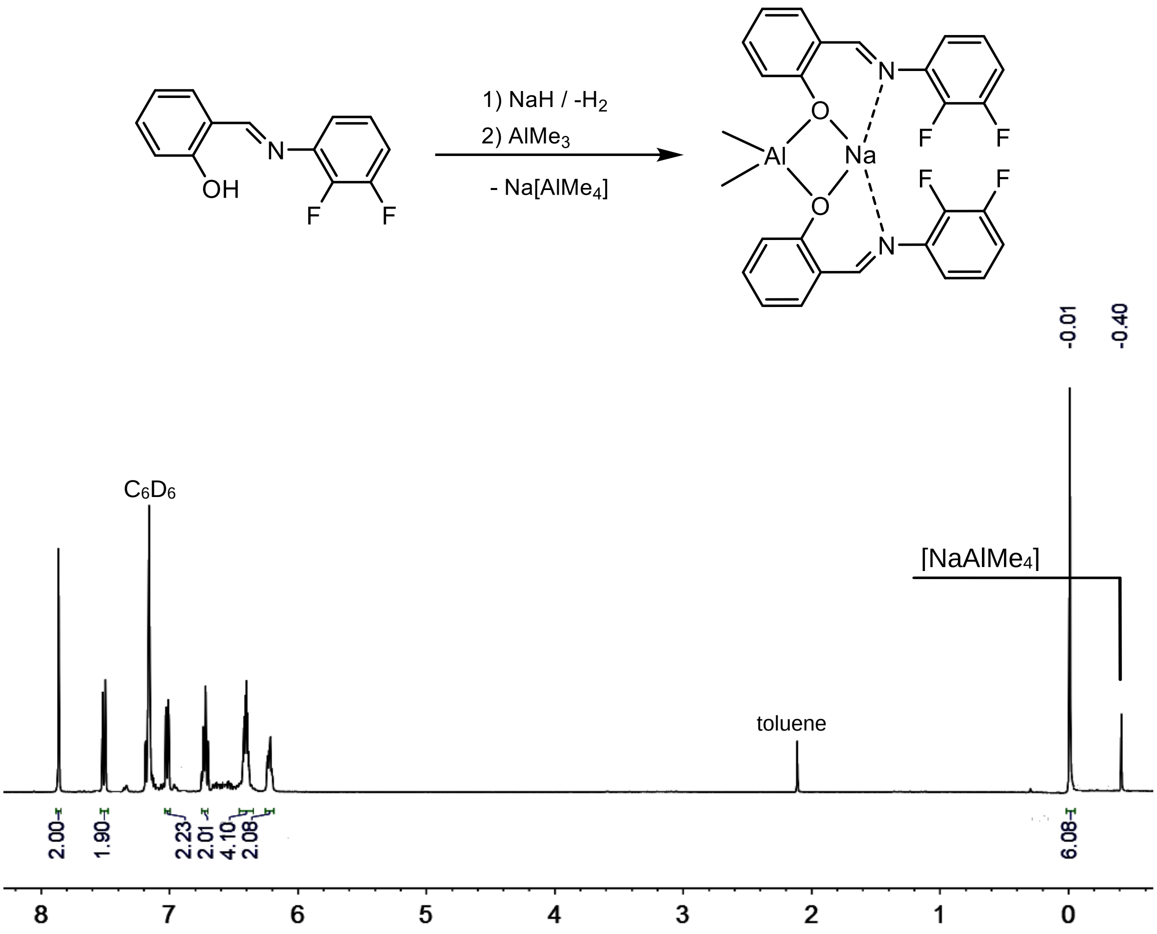

Figure S2 ^{1}H NMR spectrum of the stoichiometric reaction in a NMR tube with a 1:1:1 ratio ([HL]/[M]/[Al]), and recorded in C_6D_6 at room temperature.

3. ^{1}H NMR Spectra of [NaL] and $AlMe_3$ reaction in a 1:2 molar ratio

- Reaction: [NaL*a*] + 2 $AlMe_3$

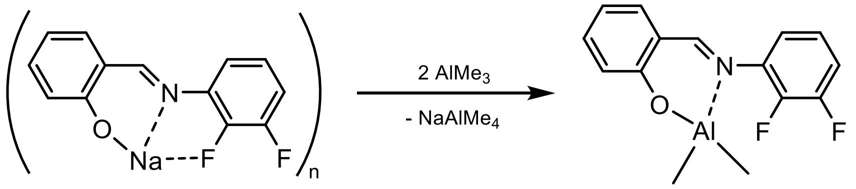

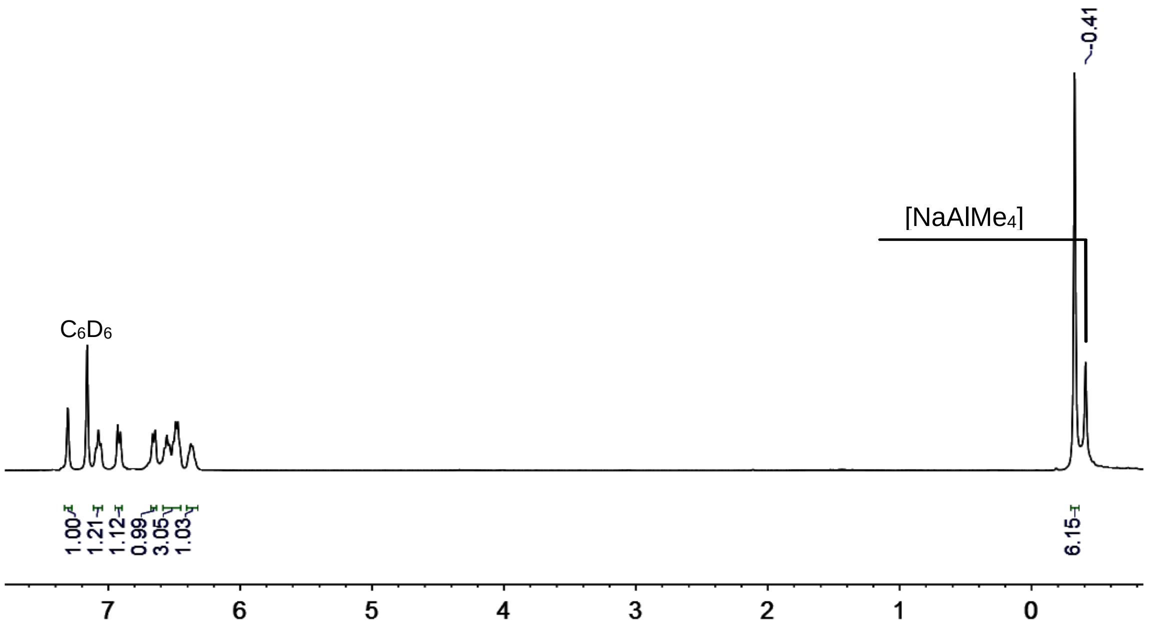

Figure S3 ^{1}H NMR spectrum of complex [$AlMe_2$(L*a*)] recorded in C_6D_6 at room temperature

4. Single-Crystal X-ray Structure Determination of (1·2C_6D_6) and 2

Table S1 Bond lengths (Å) and angles (°) for **[LiAlMe$_3$L*a*]·2(C_6D_6), (1·2C_6D_6)**

**Bond lengths (Å)**			
Li(1)-O(1)	1.982(8)	Al(1)-C(2)	1.953(5)
Li(1)-O(1)#1	1.998(8)	Al(1)-C(1)	1.994(5)
Li(1)-N(1)	2.007(9)	C(4)-C(9)	1.397(6)
Li(1)-F(1)	2.299(8)	C(4)-C(5)	1.397(6)
Li(1)-Li(1)#1	2.629(15)	C(5)-C(6)	1.352(7)
Li(1)-Al(1)	2.824(8)	C(6)-C(7)	1.380(8)
Li(1)-C(1)	2.413(4)	C(7)-C(8)	1.392(7)
N(1)-C(10)	1.280(6)	C(8)-C(9)	1.390(7)
N(1)-C(11)	1.434(6)	C(9)-C(10)	1.460(7)
O(1)-C(4)	1.363(5)	C(11)-C(16)	1.366(7)
O(1)-Al(1)	1.902(3)	C(11)-C(12)	1.392(7)
O(1)-Li(1)#1	1.998(8)	C(12)-C(13)	1.406(7)
F(1)-C(16)	1.359(5)	C(13)-C(14)	1.366(8)
F(2)-C(15)	1.336(6)	C(14)-C(15)	1.368(8)
Al(1)-C(3)	1.954(5)	C(15)-C(16)	1.381(7)
**Angles (°)**			
O(1)-Li(1)-O(1)#1	97.6(3)	C(3)-Al(1)-C(1)	109.8(2)
O(1)-Li(1)-N(1)	92.3(3)	C(2)-Al(1)-C(1)	113.4(2)
O(1)#1-Li(1)-N(1)	121.3(4)	O(1)-Al(1)-Li(1)	44.31(17)
O(1)-Li(1)-F(1)	154.7(4)	C(3)-Al(1)-Li(1)	134.1(2)
O(1)#1-Li(1)-F(1)	107.7(3)	C(2)-Al(1)-Li(1)	108.9(2)
N(1)-Li(1)-F(1)	76.0(3)	C(1)-Al(1)-Li(1)	57.0(2)
O(1)-Li(1)-Li(1)#1	49.1(2)	O(1)-C(4)-C(9)	123.4(4)
O(1)#1-Li(1)-Li(1)#1	48.5(3)	O(1)-C(4)-C(5)	118.7(4)
N(1)-Li(1)-Li(1)#1	115.2(5)	C(9)-C(4)-C(5)	117.9(4)
F(1)-Li(1)-Li(1)#1	156.2(5)	C(6)-C(5)-C(4)	121.1(4)
O(1)-Li(1)-Al(1)	42.17(17)	C(5)-C(6)-C(7)	121.9(4)
O(1)#1-Li(1)-Al(1)	114.7(3)	C(6)-C(7)-C(8)	117.6(4)
N(1)-Li(1)-Al(1)	110.9(3)	C(7)-C(8)-C(9)	121.4(4)
F(1)-Li(1)-Al(1)	121.2(3)	C(4)-C(9)-C(8)	120.1(4)
Li(1)#1-Li(1)-Al(1)	76.1(3)	C(4)-C(9)-C(10)	125.1(4)
C(10)-N(1)-C(11)	118.4(4)	C(8)-C(9)-C(10)	114.9(4)
C(10)-N(1)-Li(1)	125.6(4)	N(1)-C(10)-C(9)	126.3(4)
C(11)-N(1)-Li(1)	116.1(3)	C(16)-C(11)-C(12)	118.6(4)
C(4)-O(1)-Al(1)	116.6(2)	C(16)-C(11)-N(1)	116.3(4)
C(4)-O(1)-Li(1)	126.9(3)	C(12)-C(11)-N(1)	125.1(4)
Al(1)-O(1)-Li(1)	93.5(3)	C(11)-C(12)-C(13)	119.1(5)
C(4)-O(1)-Li(1)#1	113.6(3)	C(14)-C(13)-C(12)	121.0(5)
Al(1)-O(1)-Li(1)#1	119.0(3)	C(15)-C(14)-C(13)	118.7(5)
Li(1)-O(1)-Li(1)#1	82.4(3)	F(2)-C(15)-C(14)	121.0(4)

C(16)-F(1)-Li(1)	107.7(3)	F(2)-C(15)-C(16)	118.6(4)
O(1)-Al(1)-C(3)	111.33(19)	C(14)-C(15)-C(16)	120.4(5)
O(1)-Al(1)-C(2)	105.49(19)	F(1)-C(16)-C(11)	119.4(4)
C(3)-Al(1)-C(2)	116.0(2)	F(1)-C(16)-C(15)	118.4(4)
O(1)-Al(1)-C(1)	99.43(17)	C(11)-C(16)-C(15)	122.2(4)

Table S2 Bond lengths (Å) and angles (°) for **[NaAlMe$_3$L*a*], (2)**

Bond lengths (Å)			
Na(1)-O(1)	2.3254(18)	O(1)-Na(1)#1	2.3658(19)
Na(1)-O(1)#1	2.3657(19)	F(1)-C(16)	1.357(3)
Na(1)-N(1)	2.389(2)	F(2)-C(15)	1.347(3)
Na(1)-F(1)	2.4608(18)	C(4)-C(5)	1.395(3)
Na(1)-C(3)	2.737(6)	C(4)-C(9)	1.405(4)
Na(1)-C(4)#1	2.970(3)	C(4)-Na(1)#1	2.970(3)
Na(1)-C(5)#1	3.066(3)	C(5)-C(6)	1.385(4)
Na(1)-Al(1)	3.1940(13)	C(5)-Na(1)#1	3.066(3)
Na(1)-Na(1)#1	3.334(2)	C(6)-C(7)	1.375(4)
Na(1)-Al(1)#1	3.5005(14)	C(7)-C(8)	1.378(4)
Al(1)-O(1)	1.8621(19)	C(8)-C(9)	1.394(3)
Al(1)-C(2)	1.965(3)	C(9)-C(10)	1.468(4)
Al(1)-C(1)	1.977(4)	C(11)-C(16)	1.380(4)
Al(1)-C(3)	1.981(4)	C(11)-C(12)	1.394(4)
Al(1)-Na(1)#1	3.5004(14)	C(12)-C(13)	1.378(4)
N(1)-C(10)	1.275(3)	C(13)-C(14)	1.374(4)
N(1)-C(11)	1.416(3)	C(14)-C(15)	1.369(4)
O(1)-C(4)	1.349(3)	C(15)-C(16)	1.369(4)
**Angles (°)**			
O(1)-Na(1)-O(1)#1	89.41(6)	C(1)-Al(1)-C(3)	110.7(2)
O(1)-Na(1)-N(1)	76.05(7)	O(1)-Al(1)-Na(1)	46.01(6)
O(1)#1-Na(1)-N(1)	146.60(9)	C(2)-Al(1)-Na(1)	108.80(10)
O(1)-Na(1)-F(1)	142.63(7)	C(1)-Al(1)-Na(1)	134.23(15)
O(1)#1-Na(1)-F(1)	125.64(7)	C(3)-Al(1)-Na(1)	58.39(16)
N(1)-Na(1)-F(1)	67.32(6)	O(1)-Al(1)-Na(1)#1	39.05(5)
O(1)-Na(1)-C(3)	71.27(11)	C(2)-Al(1)-Na(1)#1	143.81(10)
O(1)#1-Na(1)-C(3)	95.30(11)	C(1)-Al(1)-Na(1)#1	76.81(13)
N(1)-Na(1)-C(3)	107.75(10)	C(3)-Al(1)-Na(1)#1	88.10(12)
F(1)-Na(1)-C(3)	112.62(13)	Na(1)-Al(1)-Na(1)#1	59.53(3)
O(1)-Na(1)-C(4)#1	105.01(7)	C(10)-N(1)-C(11)	117.3(2)
O(1)#1-Na(1)-C(4)#1	26.31(6)	C(10)-N(1)-Na(1)	123.94(18)
N(1)-Na(1)-C(4)#1	171.28(9)	C(11)-N(1)-Na(1)	116.96(16)
F(1)-Na(1)-C(4)#1	112.32(7)	C(4)-O(1)-Al(1)	122.80(15)
C(3)-Na(1)-C(4)#1	80.60(10)	C(4)-O(1)-Na(1)	126.01(15)
O(1)-Na(1)-C(5)#1	130.48(7)	Al(1)-O(1)-Na(1)	98.81(8)
O(1)#1-Na(1)-C(5)#1	49.56(6)	C(4)-O(1)-Na(1)#1	102.68(14)

N(1)-Na(1)-C(5)#1	153.47(8)	Al(1)-O(1)-Na(1)#1	111.22(8)
F(1)-Na(1)-C(5)#1	86.34(7)	Na(1)-O(1)-Na(1)#1	90.59(6)
C(3)-Na(1)-C(5)#1	84.68(10)	C(16)-F(1)-Na(1)	115.88(14)
C(4)#1-Na(1)-C(5)#1	26.67(7)	Al(1)-C(3)-Na(1)	83.56(16)
O(1)-Na(1)-Al(1)	35.18(5)	O(1)-C(4)-C(5)	118.9(2)
O(1)#1-Na(1)-Al(1)	101.74(5)	O(1)-C(4)-C(9)	122.5(2)
N(1)-Na(1)-Al(1)	83.59(6)	C(5)-C(4)-C(9)	118.5(2)
F(1)-Na(1)-Al(1)	129.25(6)	O(1)-C(4)-Na(1)#1	51.01(11)
C(3)-Na(1)-Al(1)	38.05(9)	C(5)-C(4)-Na(1)#1	80.51(15)
C(4)#1-Na(1)-Al(1)	102.31(6)	C(9)-C(4)-Na(1)#1	140.81(16)
C(5)#1-Na(1)-Al(1)	117.56(7)	C(6)-C(5)-C(4)	121.2(3)
O(1)-Na(1)-Na(1)#1	45.19(5)	C(6)-C(5)-Na(1)#1	141.05(19)
O(1)#1-Na(1)-Na(1)#1	44.22(4)	C(4)-C(5)-Na(1)#1	72.82(14)
N(1)-Na(1)-Na(1)#1	115.10(7)	C(7)-C(6)-C(5)	120.2(3)
F(1)-Na(1)-Na(1)#1	165.40(8)	C(6)-C(7)-C(8)	119.2(3)
C(3)-Na(1)-Na(1)#1	80.89(12)	C(7)-C(8)-C(9)	122.0(3)
C(4)#1-Na(1)-Na(1)#1	62.91(6)	C(8)-C(9)-C(4)	118.8(2)
C(5)#1-Na(1)-Na(1)#1	89.57(6)	C(8)-C(9)-C(10)	115.8(2)
Al(1)-Na(1)-Na(1)#1	64.81(4)	C(4)-C(9)-C(10)	125.4(2)
O(1)-Na(1)-Al(1)#1	94.28(6)	N(1)-C(10)-C(9)	127.0(2)
O(1)#1-Na(1)-Al(1)#1	29.73(5)	C(16)-C(11)-C(12)	117.0(2)
N(1)-Na(1)-Al(1)#1	120.39(7)	C(16)-C(11)-N(1)	117.6(2)
F(1)-Na(1)-Al(1)#1	110.15(6)	C(12)-C(11)-N(1)	125.2(2)
C(3)-Na(1)-Al(1)#1	124.59(11)	C(13)-C(12)-C(11)	120.5(3)
C(4)#1-Na(1)-Al(1)#1	51.08(5)	C(14)-C(13)-C(12)	121.5(3)
C(5)#1-Na(1)-Al(1)#1	64.58(6)	C(15)-C(14)-C(13)	118.0(3)
Al(1)-Na(1)-Al(1)#1	120.47(3)	F(2)-C(15)-C(16)	118.6(3)
Na(1)#1-Na(1)-Al(1)#1	55.66(3)	F(2)-C(15)-C(14)	120.3(3)
O(1)-Al(1)-C(2)	106.64(11)	C(16)-C(15)-C(14)	121.0(3)
O(1)-Al(1)-C(1)	106.14(14)	F(1)-C(16)-C(15)	119.5(2)
C(2)-Al(1)-C(1)	114.77(18)	F(1)-C(16)-C(11)	118.6(2)
O(1)-Al(1)-C(3)	101.10(15)	C(15)-C(16)-C(11)	121.9(3)
C(2)-Al(1)-C(3)	115.92(17)		
